# Mucocele of the appendix: what to expect

**DOI:** 10.1590/0100-3984.2021.0075

**Published:** 2022

**Authors:** Sofia Frade Santos, Mariana Horta, Filipa Rosa, Miguel Rito, Teresa Margarida Cunha

**Affiliations:** 1 Instituto Português de Oncologia de Lisboa Francisco Gentil (IPOLFG), Lisboa, Portugal.; 2 Instituto de Anatomia, Faculdade de Medicina, Universidade de Lisboa, Lisboa, Portugal.

**Keywords:** Mucocele/diagnostic imaging, Appendiceal neoplasms/diagnostic imaging, Adenocarcinoma, mucinous/diagnostic imaging, Cystadenoma, mucinous/diagnostic imaging, Pseudomyxoma peritonei/etiology, Mucocele/diagnóstico por imagem, Neoplasias do apêndice/diagnóstico por imagem, Adenocarcinoma mucinoso/diagnóstico por imagem, Cistadenoma mucinoso/diagnóstico por imagem, Pseudomixoma peritoneal/etiologia

## Abstract

Mucoceles of the appendix are rare and can have quite variable imaging and clinical presentations, sometimes mimicking an adnexal mass. The underlying cause can be neoplastic or non-neoplastic. The typical imaging appearance of a mucocele of the appendix is that of a cystic structure with a tubular morphology. This structure is defined by having a blind-ending and being contiguous with the cecum. Radiologists should be familiar with key anatomical landmarks and with the various imaging features of mucoceles of the appendix, in order to provide a meaningful differential diagnosis of a lesion in the right lower abdominal quadrant. In addition, a neoplastic mucocele can rupture, resulting in pseudomyxoma peritonei, which will change the prognosis dramatically. Therefore, prompt diagnostic imaging is crucial.

## Abstract (pt)

Mucoceles do apêndice são raras e podem ter uma apresentação clínica e imagiológica bastante variável, por vezes mimetizando patologia anexial. As causas subjacentes podem ser neoplásicas ou não neoplásicas. O aspecto de imagem típico de mucoceles do apêndice é o de uma estrutura de natureza cística com morfologia tubular. Esta estrutura deverá terminar “em fundo cego” e ser contígua com o ceco. Os radiologistas devem estar familiarizados com os pontos anatômicos de referência e com as diferentes características imagiológicas de mucoceles do apêndice, de modo a fornecer um adequado diagnóstico diferencial de uma lesão localizada no quadrante abdominal inferior direito. Para além disso, uma mucocele neoplásica pode sofrer ruptura, resultando em pseudomixoma peritoneal, o que altera drasticamente o prognóstico. Assim, o diagnóstico por imagem em tempo útil é crucial.

## INTRODUCTION

Mucoceles of the appendix are rare, being detected in only 0.1-0.7% of appendiceal specimens^(1)^. They typically occur in patients aged 50 to 60 years and are more common in women^(2)^. They are often found incidentally, as many patients are asymptomatic or present with non-specific symptoms^(1-3)^. The most common symptom is pain in the right lower quadrant. Therefore, they may mimic, clinically and radiologically, an adnexal mass or acute/subacute inflammation of the appendix^(4)^.

Perforation of a mucocele of the appendix can occur spontaneously or intra-operatively. This may lead to the spread of mucin, epithelial cells or both, throughout the peritoneal cavity, resulting in pseudomyxoma peritonei (PMP), which can cause life-threatening complications^(1,4,5)^. Therefore, prompt diagnostic imaging and surgical treatment are crucial.

This article reviews the imaging features of mucoceles of the appendix, as well as the most recent World Health Organization classification of epithelial tumours of the appendix, underscoring the importance of establishing an accurate differential diagnosis. From the database of our cancer centre, we selected some of the most representative cases of mucocele of the appendix seen over the last five years, in order to illustrate their imaging features. We included cases of patients that were assessed pathologically and by various imaging techniques, such as ultrasound (Logiq 9; GE Healthcare, Wauwatosa, WI, USA), computed tomography (CT) in a dual-source scanner (Somatom; Siemens Healthineers, Erlangen, Germany) and magnetic resonance imaging (MRI) in 1.5-T and 3.0-T scanners (Intera and Ingenia, respectively; Philips Medical Systems, Andover, MA, USA).

## CAUSES AND ANATOMOPATHOLOGICAL CLASSIFICATION

Macroscopically, a mucocele of the appendix manifests as an appendix that is abnormally distended by mucus, and the mucocele can have different underlying aetiologies^(2,4,5)^. In fact, mucoceles of the appendix can have a non-neoplastic cause (such as a simple retention cyst) or be produced by a mucin-secreting epithelial neoplasm^(5)^. When the underlying cause is neoplastic, there can be unregulated mucin production with consequent cystic dilation. That poses a risk of perforation, which would lead to PMP. There is no such risk of disease spread in cases with non-neoplastic causes, as there are no epithelial cells in the mucus^(1,2,6,7)^.

There has been substantial controversy about the pathological classification of and nomenclature related to epithelial tumours of the appendix. According to the World Health Organization pathological classification, updated in 2019^(8,9)^, the neoplastic causes of a mucocele include serrated lesions/polyps, low-grade appendiceal mucinous neoplasms (LAMNs), high-grade appendiceal mucinous neoplasms (HAMNs) and mucinous adenocarcinomas. Serrated lesions/polyps are mucosal polyps characterised by a saw-toothed or stellate crypt lumen. Such lesions include hyperplastic polyps and sessile serrated lesions, without or with dysplasia. A lesion growing beyond the mucosa, with pushing borders, is classified as a LAMN or a HAMN. A LAMN has low-grade cytologic atypia and features such as loss of the muscularis mucosae, fibrosis of the submucosa, rupture of the appendix and mucin or cells outside the appendix. A HAMN has all of the architectural features of a LAMN but features high-grade cytologic atypia. The term “mucinous adenocarcinoma” should be reserved for lesions with frankly infiltrative invasion^(8,9)^. The term “cystadenoma” is considered an outdated term, and its use is therefore no longer recommended. According to data currently available in the literature^(8,9)^, any lesion referred to as a mucinous “cystadenoma” is likely a LAMN.

## IMAGING FINDINGS

### Ultrasound

On ultrasound, a mucocele of the appendix appears as an ovoid or pear-shaped cystic mass in the right lower quadrant, where the appendix is usually located. Although the well-known “onion-skin” appearance (internal concentric echogenic layers of mucin) is considered typical, the internal echotexture of mucoceles of the appendix varies. Dystrophic mural calcifications can produce acoustic shadowing, a feature that is seen in less than half of all cases^(2,5)^.

### CT

**Figure 1:**
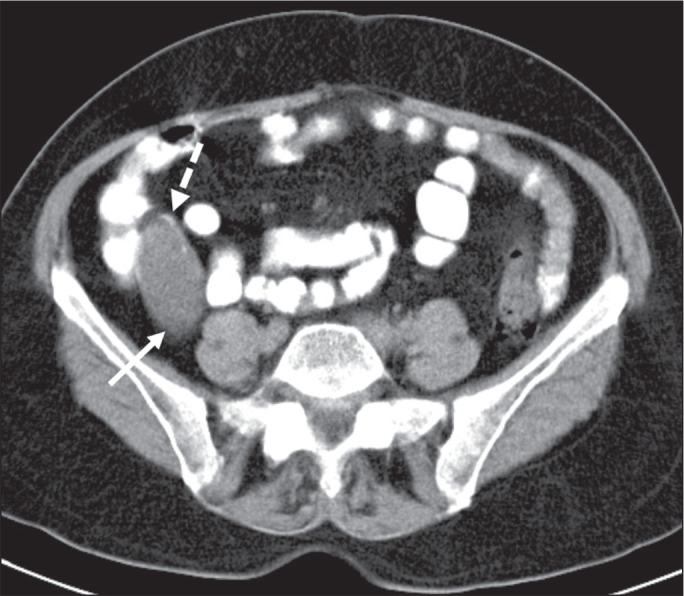
Mucocele of the appendix: an incidentaloma in a 78-year-old woman. Axial CT scan of the abdomen and pelvis, showing a hypodense ovoid structure (solid arrow) in close proximity to the ileocecal valve, measuring 2.8 cm in diameter. Note also the curvilinear mural calcifications (dashed arrow).

On CT, the appearance of a mucocele of the appendix is that of a blind-ending tubular structure that is contiguous with the cecum and filled with homogeneous, low-attenuation content^(2)^. As illustrated in Figure 1, curvilinear mural calcifications constitute a distinctive feature of mucoceles of the appendix, although they are not always present^(5)^.

### MRI

On MRI, the interior of a mucocele of the appendix usually shows a signal characteristic of simple fluids. However, that signal may vary according to the protein content^(2,5)^.

### Aetiology

**Figure 2:**
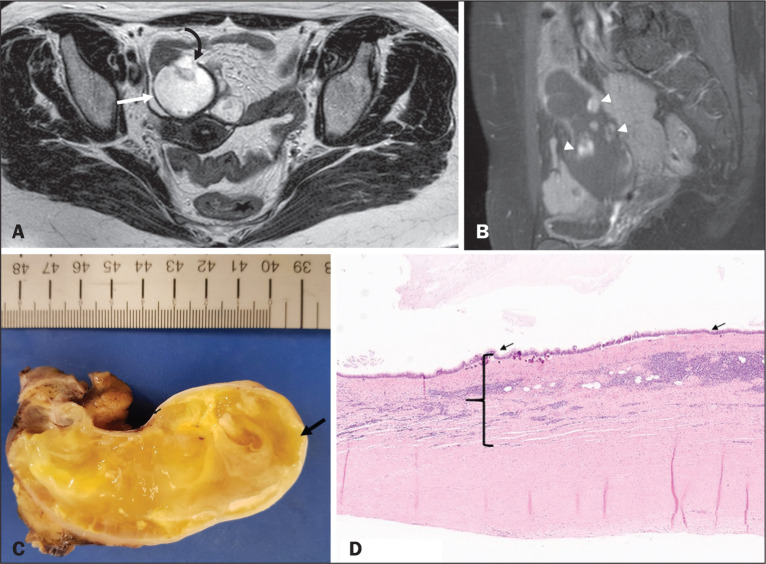
LAMN. Axial T2-weighted MRI sequence (A) and gadolinium contrast-enhanced sagittal T1-weighted MRI sequence with fat suppression (B), showing a mucocele of the appendix (white arrow in A), measuring 8.6 cm at its maximum longitudinal diameter. Solid parietal nodules (arrowheads in B) are noted, the largest measuring 8 mm, a feature that is suggestive of malignancy. A small suspected point of rupture is seen anteriorly (curved arrow in A). C: Gross specimen obtained by appendectomy with partial resection of the cecum, measuring 9 cm in length and 4 cm at its maximum diameter. On cross-section, the appendiceal lumen is markedly distended by an abundant translucent, yellowish-white viscous content (arrow), with three luminous projections, each measuring less than 1 cm. The surface has a 1-cm focus of rupture. D: Replacement of the normal colonic epithelium by a monolayer of cytologically bland mucinous epithelium (arrow). The lymphoid tissue of the appendix is absent, and there is subepithelial fibrosis and calcifications ({), the tumour showing a broad pushing border (haematoxylin and eosin; magnification, ×25). The final histopathologic diagnosis was LAMN.

**Figure 3:**
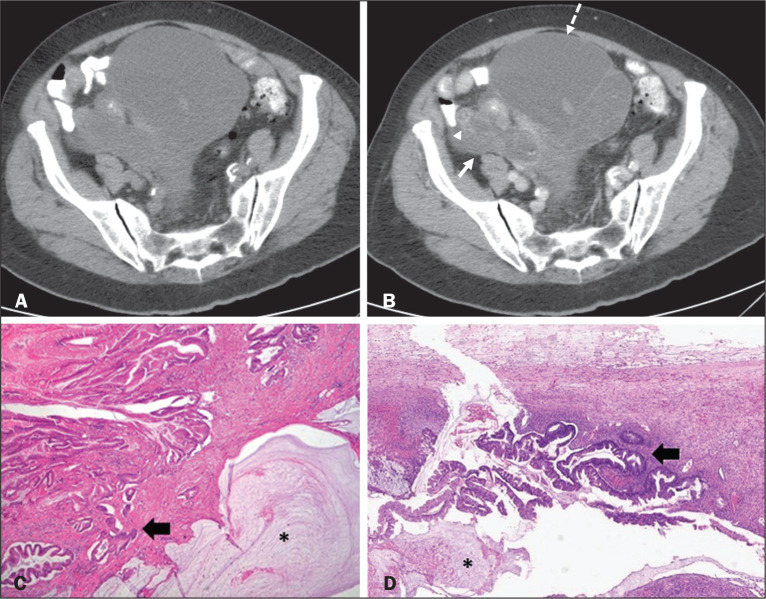
Mucinous adenocarcinoma of the appendix with ovarian involvement. A,B: Axial CT scans, before and after intravenous contrast administration, showing a tubular structure corresponding to a distended appendix (solid arrow), with irregular wall thickening and enhancing mural nodularity (arrowhead), features that are suggestive of malignancy. A large, predominantly cystic lesion is also seen, apparently in the right ovary (dashed arrow). C,D: The final histopathologic diagnosis was a mucinous adenocarcinoma of the appendix with metastasis to the right ovary (haematoxylin and eosin; magnification, ×40). Atypical irregular glands (arrow) infiltrating the wall of the appendix (C) with a desmoplastic stromal response. Extracellular mucin (asterisk) composing more than 50% of the tumour. A similar neoplasm can be seen in the ovary (D), with a CK20+/CK7- phenotype, consistent with colorectal origin.

Although differentiating between benign and malignant causes of a mucocele of the appendix is relevant for the prognosis (Figures 2 and 3), making that distinction preoperatively continues to be a challenge^(2)^. Mural nodularity and irregular wall thickening have been associated with malignancy. Variables such as lesion diameter, attenuation of internal content, presence of internal septations and wall calcifications are not considered helpful in distinguishing between malignant and benign mucoceles^(3,5)^. However, benign retention cysts usually do not exceed 2 cm in their short-axis diameter^(2)^.

### PMP

**Figure 4:**
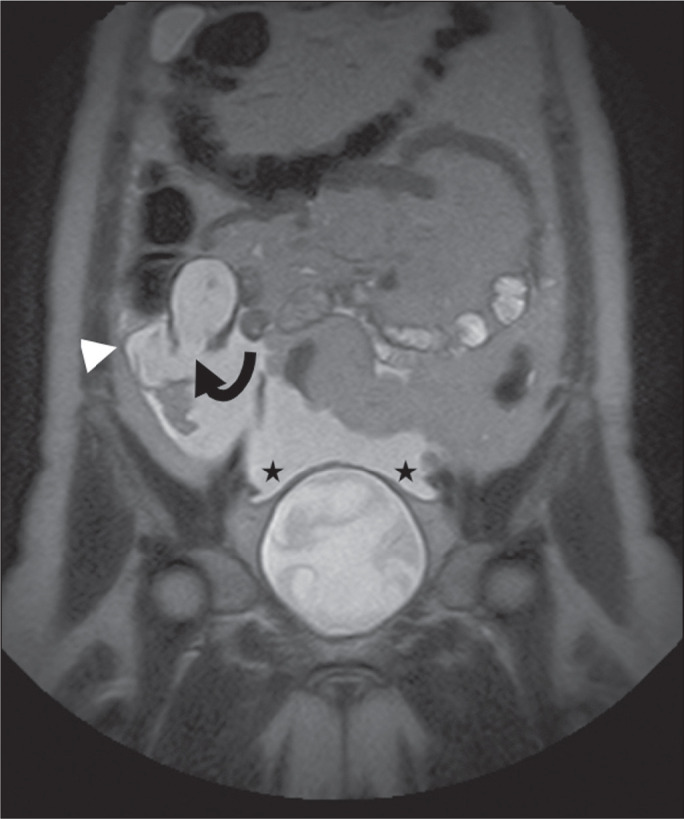
Mucocele of the appendix. Coronal T2-weighted MRI sequence showing a ruptured mucocele of the appendix (curved arrow), together with PMP, with peri-appendicular fluid (arrowhead) and fluid in the pelvic cavity (stars).

**Figure 5:**
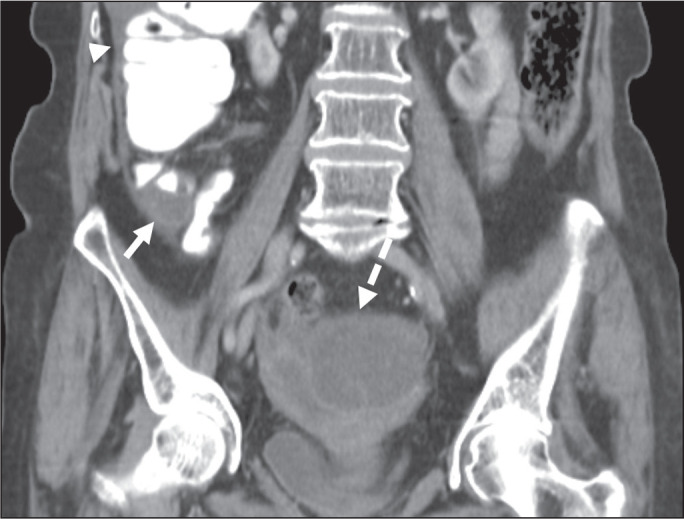
LAMN with PMP and ovarian involvement. Contrast-enhanced coronal CT scan showing a mucocele of the appendix (solid arrow), with PMP involving the right paracolic gutter (arrowhead). Note also the cystic tumour in the left ovary (dashed arrow). Histological analysis after surgery revealed two mucinous tumours (a LAMN in the appendix and another in the left ovary), which overlapped morphologically. It is likely that the primary neoplasm arose in the appendix, subsequently extending to the ovary and peritoneum.

The rupture of a mucinous tumour leads to PMP^(5)^, a clinical syndrome that manifests as intraperitoneal accumulation of mucus. It almost always arises from a perforated mucinous neoplasm of the appendix (Figures 4 and 5). Less common sources include mucinous tumours of the ovary, colon, urachus and pancreas^(5,10)^.

The “redistribution phenomenon” characterises the pattern of peritoneal involvement of PMP, reflecting the usual pathway of the flow of peritoneal fluid. The mucus, cells or both are preferentially “redistributed” within the peritoneal cavity to sites of fluid reabsorption, following a predictable route. They typically accumulate in the pelvis, paracolic gutters, great omentum and perihepatic spaces (subphrenic spaces and Morison’s pouch), initially sparing the mesentery of the small intestine^(5)^.

**Figure 6:**
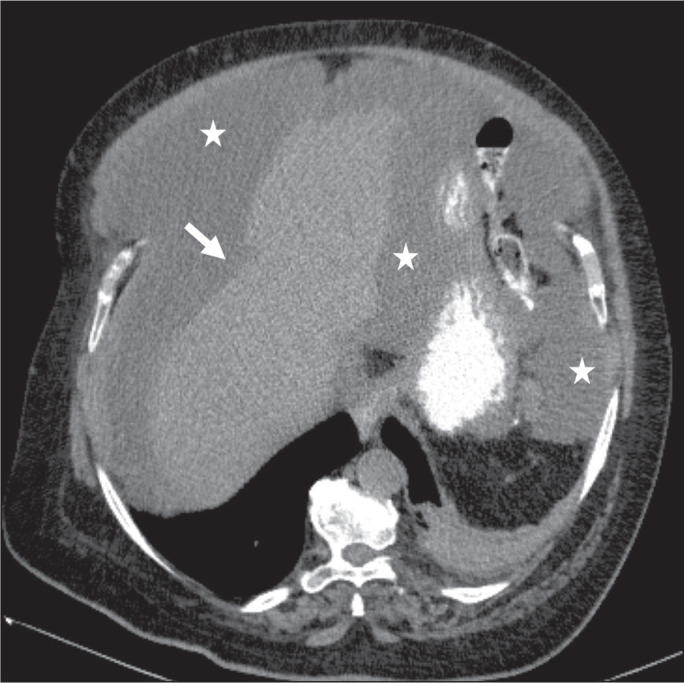
PMP. Axial CT scan shows the characteristic findings associated with PMP. Note the ascites (stars) and the typical scalloping of the surface of the liver, which was most pronounced in segment VIII (arrow).

If a mucocele of the appendix is identified, an attempt to detect extraluminal mucin should be made^(1,2,5)^. On CT scans of patients with PMP, the following features are usually seen: mucinous ascites (which can become large in volume, with or without septa); peritoneal soft-tissue implants; “omental caking”; and ovarian involvement. Mucinous implants manifest as low-attenuation nodules that can contain coarse calcifications. Scalloping of the surface of solid abdominal organs, such as the liver (Figure 6), is quite typical and represents a characteristic mass effect of mucinous implants^(5,6,10)^. In a number of studies^(5,6)^, it has been shown that MRI has a higher sensitivity than does CT for the detection of PMP-related implants (84% vs. 54%).

## DIFFERENTIAL DIAGNOSIS

**Figure 7:**
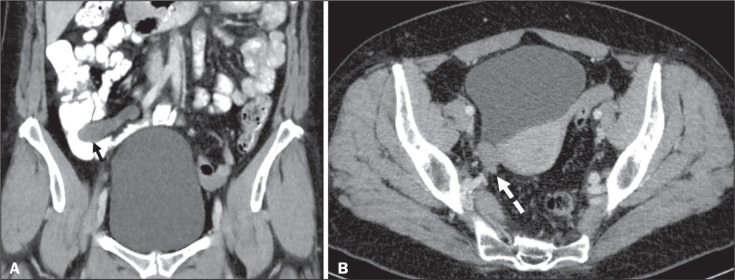
Mucocele of the appendix. Contrast-enhanced coronal and axial CT scans (A and B, respectively), showing the typical appearance of a mucocele of the appendix and the relationships with the surrounding anatomical landmarks. A blind-ending tubular cystic structure can be seen at the expected location within the appendix (arrow in A). The individualisation of the right ovary separately from the above-mentioned tubular structure is highlighted (arrow in B).

**Figure 8:**
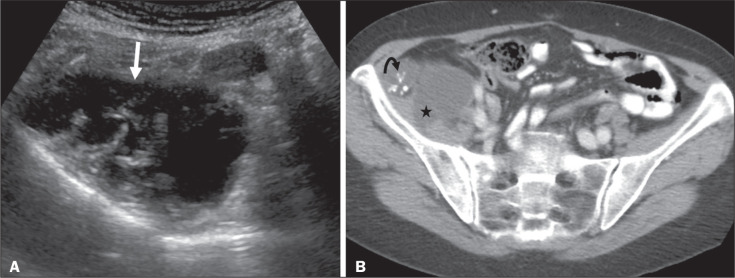
LAMN with an atypical presentation. A: Ultrasound image shows a multilocular cystic tumour (arrow) in the right iliac fossa. B: Contrast-enhanced axial CT scan showing infiltration of the right iliac muscle (star) by a tumour, representing a ruptured mucocele of the appendix, in an extra-peritoneal location. Note the cluster of calcifications within the tumour (curved arrow) and the fact that the cortex of the iliac bone is intact. The right iliopsoas muscle was excised. In the surgical specimen, cystic areas filled with mucinous content were observed. The final histological diagnosis was a ruptured LAMN.

**Figure 9:**
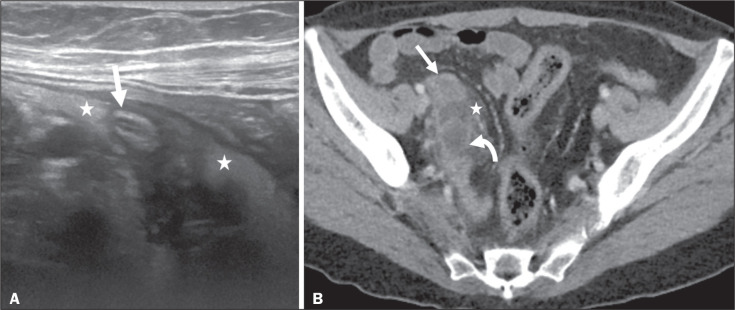
Acute appendicitis and a simple retention cyst. A,B: Imaging evaluation of a patient with acute pain in the right lower quadrant, showing an appendix (solid arrow) with an increased caliber (16 mm) and signs of peri-appendiceal inflammation (stars) that manifest as hyperechoic, non-compressible fat on ultrasound (A) and densification of the adjacent fat on an axial CT scan (B). A hypodense/cystic area (curved arrow) is observed at its distal end, suggesting a mucocele or a peri-appendiceal fluid collection. A retrospective review of a CT study performed one month before (not shown) revealed that a tiny cystic structure was already visible at the tip of the appendix. The appendectomy specimen showed a thickened appendix, and the histological diagnosis was acute appendicitis accompanied by peritonitis and epithelial hyperplasia, without neoplastic tissue (a simple retention cyst).

There is a broad spectrum of conditions (Table 1), especially those arising in the right ovary, that can mimic an appendiceal pathology^(4)^. To provide a meaningful differential diagnosis, the radiologist should not only characterise the morphology and content of the lesion but also assess its relationship with key anatomical landmarks in the abdomen and pelvis in order to determine its origin^(4)^. The relationship between the appendiceal mass and the cecal pole should be determined, and the right ovary should be recognised^(5)^, as depicted in Figures 7 and 8. A comprehensive imaging evaluation, especially with cross-sectional methods (CT or MRI), together with clinical and laboratory data, usually provides clues to the diagnosis (Table 2). It should be borne in mind that it may be difficult to distinguish between a mucocele of the appendix and acute appendicitis, as well as that the two may even coexist, resulting in inflammation in the surrounding area (Figure 9).

**Table 1:** Differential diagnoses of a lesion in the right lower abdominal quadrant suspected of being a mucocele of the appendix.

Differential diagnosis
Neoplastic
Right cystic ovarian tumour (mucinous cystadenoma, mucinous borderline tumour, mucinous carcinoma, serous cystadenoma, serous borderline tumour, serous carcinoma, and cystadenofibroma)
Right paraovarian serous cystadenoma
Non-neoplastic
Acute/subacute appendicitis*
Peri-appendiceal abscess
Right tubo-ovarian abscess
Right hydrosalpinx
Peritoneal inclusion cyst
Lymphocele
Enteric duplication cyst

* Appendicitis and mucocele of the appendix may coexist

**Table 2:** Main imaging features of a mucocele of the appendix.

Key imaging feature(s)	Imaging method(s)
Ovoid or pear-shaped cystic mass	Ultrasound/CT/MRI
Lesion arising from the cecum or separated from the right ovary	Ultrasound/CT/MRI
“Onion-skin” appearance	Ultrasound
Mural calcifications	Ultrasound/CT
Findings associated with malignancy: mural nodularity and irregular wall thickening	CT/MRI

## CONCLUSION

Appendiceal mucoceles are rare and can pose a diagnostic challenge, especially because they may be mistaken for a cystic lesion of the right ovary. Radiologists should be aware that the underlying cause of a mucocele of the appendix may range from a simple retention cyst to a malignant tumour. Although an imaging evaluation still cannot reliably predict the underlying histology, there are a few imaging clues that suggest associated malignancy, such as mural nodularity and irregular wall thickening. It is of paramount importance that the diagnosis is suspected when the lesion is still confined to the appendix.
